# Spontaneous Coronary Artery Dissection Associated with Anal Cancer Management with Fluorouracil and Radiotherapy

**DOI:** 10.7759/cureus.4979

**Published:** 2019-06-23

**Authors:** Kimberly Hart, Suketu Patel, Joshua Kovoor

**Affiliations:** 1 Radiation Oncology, The Karmanos Cancer Center's Gershenson Radiation Oncology Center, Wayne State University School of Medicine, Detroit, USA

**Keywords:** radiotherapy, anal cancer, anal squamous cell carcinoma, 5-fluorouracil, spontaneous coronary artery dissection, scad

## Abstract

Spontaneous coronary artery dissection (SCAD) is thought to be a rare condition that is hard to predict due to the lack of easily identifiable warning signs. We report the case of a 49-year-old woman with a locally advanced Stage IIIB anal squamous cell carcinoma who presented with chest pain and a positive stress test, ST elevations in her inferior echocardiogram leads, and induced chest pain with exercise without heart perfusion defects. Coronary catheterization revealed a right coronary artery dissection, which led to the diagnosis of SCAD. Our patient was diagnosed while undergoing a combination treatment of fluorouracil (5-FU), mitomycin, and pelvic radiotherapy. We reviewed the current literature and update the etiologies that have been proposed since the publication of this case report.

## Introduction

Spontaneous coronary artery dissection (SCAD) describes a phenomenon where layers of the coronary arterial wall disrupt and fill with blood without evidence of atherosclerotic disease. This can interrupt blood flow to the cardiac muscle, resulting in acute coronary syndromes, including myocardial infarction and sudden death [1].

The standard treatment for squamous cell anal cancer after 1980 has been chemoradiotherapy (CRT). The Nigro trials showed there was greater local control of the malignancy with CRT versus radiotherapy (RT) alone [2]. Chemoradiotherapy also allows surgical procedures that resect part of the colon to be avoided in the majority of patients and withheld to only a minority of patients that have recurrent or persistent disease after CRT [3]. Our patient received mitomycin-C, fluorouracil (5-FU), and pelvic radiotherapy, which increased risk factors that cause mechanical stress on heart vessels. We report the rare case of SCAD secondary to the treatment of anal cancer.

## Case presentation

Patient profile

**Figure 1:**
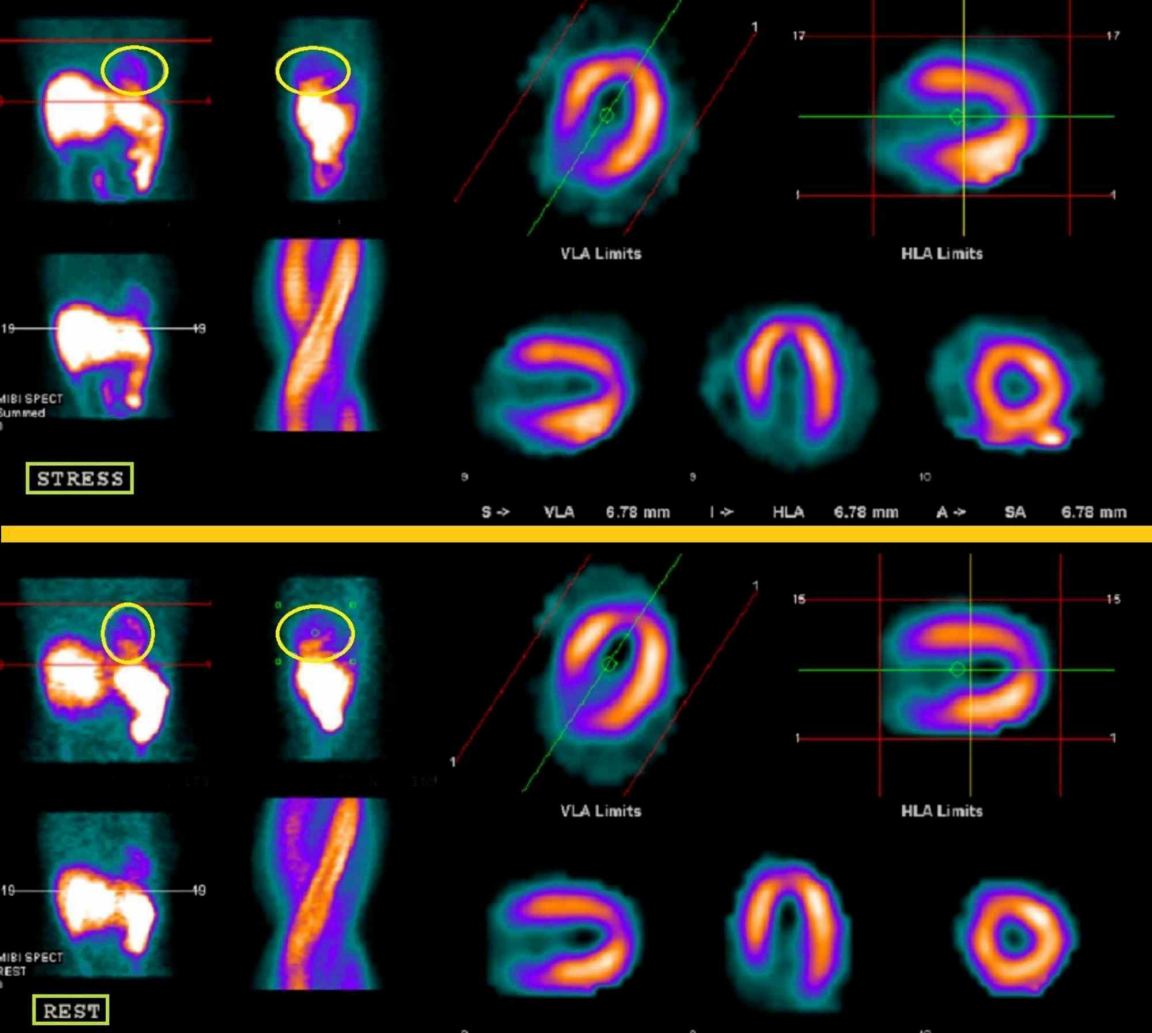
Myocardial Perfusion Imaging of Spontaneous Coronary Artery Dissection (SCAD) The yellow circled areas on the scans in the regions labeled STRESS and REST in the figure highlight the region of the heart hypoperfused by the SCAD. The region of the figure labeled STRESS illustrates the myocardial perfusion while the patient's heart is under stress. The region of the figure labeled REST illustrates the myocardial perfusion while the patient is at rest. Both sets of scans show how the hypoperfused region, in the yellow circles, does not change with activity. HLA: horizontal long axis; VLA: vertical long axis

This study involved a 49-year-old woman who presented with a Stage IIIB locally advanced anal squamous cell carcinoma with no prior cardiovascular risk factors. Her treatment regimen consisted of 5-FU (7,200 mg/96 hours), mitomycin (18 mg), and 5,400 cGy delivered by RapidArc® intensity-modulated radiation therapy (IMRT) (Varian Medical Systems, Inc., Palo Alto, CA). She presented with sharp left-sided chest pain with exertional dyspnea and nausea four days after receiving chemotherapy and two cycles of RT for a total dose of 360 cGy. After treatment, she had protracted emesis and sharp retrosternal pain that radiated to the back, which increased with deep inspiration and cough. The pain was exaggerated by changing position and ameliorated with rest. She was admitted and treated with catheter angiography for myocardial infarction (MI). The causative lesion was coronary artery dissection evaluated by myocardial perfusion imaging, as shown in Figure 1, and identified using coronary catheterization which revealed an ostial right coronary artery that was 70% dissected.

After the patient was diagnosed with SCAD, she underwent catheterization with a drug-eluting stent and was placed on ticagrelor, aspirin, and pravastatin. Radiotherapy was resumed; however, during the second cycle of chemotherapy, the patient was admitted for chest pain again. She was monitored by troponin biomarkers and electrocardiograms (EKGs); however, there were no changes in cardiac function. The patient’s chest discomfort was attributed to anxiety and she was, therefore, treated with lorazepam. The patient developed a neutropenic fever of 38.7 degrees Celsius secondary to chemotherapy. The radiotherapy was stopped for 10 days secondary to the neutropenia. The SCAD continued to be monitored by the patient’s chest symptoms and by single photon emission computed tomography (SPECT) imaging. Her SPECT myocardial perfusion scan six months after the completion of her radiotherapy and chemotherapy treatment noted the left ventricle had a normal ejection fraction of 66% and there were no abnormal left ventricle wall thickenings or motions. It also revealed there were no myocardial perfusion defects.

## Discussion

A case study from 2003 described a similar presentation in which a woman receiving 5-FU chemotherapy and pelvic radiotherapy presented with hyperemesis [4]. The proposed risk factors, in that case, included 5-FU-related vasoconstrictive effects and the Valsalva stress of protracted vomiting.

Epidemiology

The true incidence of SCAD is difficult to determine. There are no biochemical markers that differentiate SCAD from other acute coronary syndromes. Although the relationship between hormonal change and SCAD is not fully understood, there is a high prevalence of SCAD in pregnancy which may be related to the rapid changes in circulating estrogen and gonadotropins [4]. Historically, SCAD was thought to be rare, but increasing use of early angiography have revealed SCAD to be more common than believed [5]. SCAD affects young to middle-aged women with 90% of the cases reported in this group [6-7]. The mean age is between 44 - 55 years old. SCAD can be triggered by events that cause increased cardiovascular stress, such as childbirth or emotional stress. Excess progesterone also leads to impaired collagen synthesis which weakens the coronary vasculature. Women on hormonal therapy would be at an increased risk for SCAD [8]. One in 10 women under 50 years old presenting with the acute coronary syndrome will be observed to have SCAD. Pregnancy-associated SCAD has been found to be a smaller proportion of events than early case series indicated [9-10].

Pathophysiology

There are two mechanisms for the pathophysiology of SCAD. The first is an accumulation of blood in the vessel wall by rupture of the vasa vasorum. The vasa vasorum are small blood vessels that travel and supply blood to the walls that make up arteries and veins. If those vasa vasorum rupture, they create a false-lumen between the walls. The second is the direct disruption of the tunica intima, the innermost wall, leading to a collection of blood between the tunica intima and tunica media. The tunica media is the muscular layer in arteries and veins. This layer is larger in arteries, so once the intima is disrupted, it instead dissects the intima rather than piercing through [8].

Precipitating factors

Our patient had two factors related to mechanical stress on the wall of the artery which could precipitate an intimal tear and subsequent hematoma formation [8]. The first was the protracted vomiting. The second was the myocardial ischemia induced by the vasoconstrictive effects of the 5-FU. The vasoconstriction induces chest pain associated with effort, rest, or a variant between the two levels of activity. It presents as a sudden onset chest pain similar to an acute heart attack once the 5-FU reaches peak systemic levels [11].

## Conclusions

SCAD is an unusual complication but one that should be monitored for in patients undergoing 5-FU treatment, especially if they have protracted emesis. The vasoconstrictive effects of 5-FU, combined with the action of emesis, risk stressing the heart and its blood vessels beyond their compliance limit. This eventually can lead to a tear in the intimal layer of a coronary vessel, leading to SCAD. We believe this was the case with our patient, who had both risk factors. If SCAD is suspected, physicians should monitor these patients with myocardial perfusion imaging to note areas of hypoperfusion and decreased myocardial activity. Overall, physicians should keep a high suspicion for SCAD if a patient undergoing 5-FU presents with heart-related symptoms.

## References

[1] Tweet MS, Kok SN, Hayes SN (2018). Spontaneous coronary artery dissection in women: what is known and what is yet to be understood. Clin Cardiol.

[2] Nigro ND, Vaitkevicius VK, Buroker T, Bradley GT, Considine B (1981). Combined therapy for cancer of the anal canal. Dis Colon Rectum.

[3] Sana S, Khan AU (2009). Clinical trials in the management of anal cancer. Clin Colon Rectal Surg.

[4] Abbott JD, Curtis JP, Murad K, Kramer HM, Remetz MS, Setaro JF, Brennan JJ (2003). Spontaneous coronary artery dissection in a woman receiving 5-fluorouracil--a case report. Angiology.

[5] Al-Hussaini A, Adlam D (2017). Spontaneous coronary artery dissection. Heart.

[6] Luong C, Starovoytov A, Heydari M, Sedlak T, Aymong E, Saw J (2017). Clinical presentation of patients with spontaneous coronary artery dissection. Catheter Cardiovasc Interv.

[7] Vanzetto G, Berger-Coz E, Barone-Rochette G (2009). Prevalence, therapeutic management and medium-term prognosis of spontaneous coronary artery dissection: results from a database of 11,605 patients. Eur J Cardiothorac Surg.

[8] Ahmed B, Creager MA (2017). Alternative causes of myocardial ischemia in women: an update on spontaneous coronary artery dissection, vasospastic angina and coronary microvascular dysfunction. Vasc Med.

[9] Hayes SN, Kim ESH, Saw J (2018). Spontaneous coronary artery dissection: current state of the science: a scientific statement from the American Heart Association. Circulation.

[10] Velusamy M, Fisherkeller M, Keenan ME, Kiernan FJ, Fram DB (2002). Spontaneous coronary artery dissection in a young woman precipitated by retching. J Invasive Cardiol.

[11] Herrmann J, Yang EH, Iliescu CA (2016). Vascular toxicities of cancer therapies: the old and the new-an evolving avenue. Circulation.

